# Melatonin Enhances the Postharvest Disease Resistance of Blueberries Fruit by Modulating the Jasmonic Acid Signaling Pathway and Phenylpropanoid Metabolites

**DOI:** 10.3389/fchem.2022.957581

**Published:** 2022-07-22

**Authors:** Guangfan Qu, Wenneng Wu, Liangjie Ba, Chao Ma, Ning Ji, Sen Cao

**Affiliations:** Food and Pharmaceutical Engineering Institute, Guiyang University, Guiyang, China

**Keywords:** blueberry, melatonin, jasmonic acid signaling pathway, phenylpropanoid metabolites, disease resistance

## Abstract

In this study, to investigate the physiological and molecular mechanisms of melatonin inhibiting the postharvest rot of blueberry fruits, blueberry fruits were dipped in 0.3 mmol L^−1^ melatonin solution for 3 min and stored at 0°C for 80 days. The results indicated that melatonin did not significantly (*p* > 0.05) inhibit the mycelial growth or spore germination of *Alternaria alternata*, *Botrytis cinerea*, and *Colletotrichum gloeosporioides*. In addition, an *in vivo* study revealed that melatonin treatment increased the enzymatic activities of phenylalanine ammonia lyase (PAL), cinnamate 4-hydroxylase (C4H), 4-coumarate-CoA ligase (4CL), cinnamyl alcohol dehydrogenase (CAD), polyphenol oxidase (PPO), and peroxidase (POD) in fruits. Furthermore, genes related to jasmonic acid synthesis were upregulated (*VaLOX*, *VaAOS*, and *VaAOC*), as were those related to pathogenesis-related proteins (*VaGLU* and *VaCHT*) and phenylpropane metabolism (*VaPAL*, *VaC4H*, *Va4CL*, *VaCAD*, *VaPPO*, and *VaPOD*), which promoted the accumulation of total phenols, flavonoids, anthocyanins, and lignin in the fruits. These results suggest that melatonin enhances the postharvest disease resistance of blueberry fruits by mediating the jasmonic acid signaling pathway and the phenylpropane pathway.

## Introduction

Blueberries (*Vaccinium* sp. in the Ericaceae family) produce small fruits that are rich in nutritional and health-promoting components such as anthocyanins, flavanols, and polyphenols, which have been suggested to have antiaging, immune-enhancing, and memory-improving functions (Gong et al., 2014; Ji N. et al., 2021.). However, blueberries are susceptible to microbial pathogens during storage and sale because of their thin skins and high moisture contents. Infestation by microbial pathogens causes rapid fruit softening, water loss, rot, and nutrient loss (Wang et al., 2010), which severely reduce the commercial value of the fruit. A wide range of microbial pathogens can cause the postharvest rot of blueberries, mainly *Alternaria alternata*, *Botrytis cinerea*, and *Colletotrichum gloeosporioides* (Wang et al., 2010). Recently, it has been reported that exogenous substances such as ethanol (Ji Y. et al., 2021), natamycin (Saito et al., 2022), sodium nitroprusside (Ge et al., 2019), and jasmonic acid methyl ester (Wang et al., 2020a) can minimize the postharvest rot of blueberries. The effects of these substances are mainly attributed to the inhibition of the growth of microbial pathogens *via* their action as fungicides or to the induction of disease resistance in fruits *via* their action as exogenous inducers.

Jasmonic acid (JA) is an important phytohormone that regulates plant growth and development and induces resistance to biotic and abiotic stresses (Liu et al., 2019). In plant defense responses, plants can not only activate the JA signaling pathway by transmitting the systemic proteins produced by pathogenic microorganisms as signal molecules to adjacent parts through apoplast and phloem but also activate JA signaling by using exogenous inducers and transmit the corresponding signal molecules to adjacent parts for defense response (Truman et al., 2007). In addition, the secondary metabolites of phenylpropane, such as total phenols, flavonoids, and lignans, are closely related to plant defense functions, and the synthesis and accumulation of these metabolites are regulated by phenylalanine ammonia lyase (PAL), cinnamate 4-hydroxylase (C4H), 4-coumarate-CoA ligase (4CL), cinnamyl alcohol dehydrogenase (CAD), polyphenol oxidase (PPO), peroxidase (POD), and other key enzymes (Ren et al., 2020; Ji N. et al., 2021.). However, on the ripening and senescence of the fruit, the activity of these enzymes gradually decreases, and the accumulation of phenylpropane metabolites slowly decreases, resulting in the loss of fruit defenses against biotic and abiotic stresses (Bennett and Wallsgrove, 1994). However, the treatment of fruits with exogenous inducers, such as salicylic acid (Zhang H. et al., 2021), terpinen-4-ol (Li et al., 2020), and sodium nitroprusside (Ge et al., 2019), can effectively regulate the enzymatic activities of phenylpropane metabolism in fruits and promote the accumulation of the secondary metabolites of phenylpropane, thus improving the postharvest disease resistance.

Melatonin (MT) is an endogenous bioactive molecule with multiple regulatory functions involved in various physiological processes, such as ripening, aging, and defense in fruits and vegetables (Li et al., 2017). In recent years, MT has been shown to not only improve the postharvest fruit quality but also enhance fruit resistance to pathogenic microorganisms (Moustafa-Farag et al., 2020). Shang et al. (2021) found that MT induction promoted the accumulation of total phenols, flavonoids, and anthocyanins in blueberry fruits and enhanced the antioxidant capacity of fruits. It was also found that the accumulation of polyphenols, flavonoids, and anthocyanins could effectively enhance the antioxidant capacity of fruits and delay the softening and senescence of fruits (Lin et al., 2018; Lin et al., 2020.). In addition, MT treatment has been shown to induce the enhanced postharvest disease resistance in jujube fruit (*Ziziphus jujuba*) (Zhang et al., 2022), cherry tomatoes (Liu et al., 2019), and grapes (Gao et al., 2020), and this is mainly attributed to the role of MT in scavenging reactive oxygen radicals, improving the antioxidant capacity, maintaining the metabolic energy balance, and promoting the accumulation of secondary metabolites of phenylpropane. MT can also mediate disease-resistance signaling pathways, such as those involving JA, NO, and salicylic acid, to improve the fruit disease resistance (Shi et al., 2015.; Liu et al., 2019.; Bhardwaj et al., 2022). However, the physiological and molecular mechanisms by which MT enhances the postharvest disease resistance in blueberries remain unclear.

Therefore, we investigated the potential physiological and molecular mechanisms by which MT maintains the blueberry quality and reduces the postharvest rot, focusing on 1) the effect of MT on the postharvest pathogenic microorganisms (*B. cinerea*, *A. alternata*, and *C. gloeosporioides*) in blueberries, 2) the effect of MT on the fruit JA signaling pathway, and 3) the effect of MT on the metabolites of phenylpropane.

## Materials and Methods

### Fruits and Treatments

Rabbiteye blueberry (*Vaccinium ashei* cv. Powder blue) fruits were harvested in July 2021 from a blueberry plantation in Majiang, China. Fully ripe fruits were selected and picked by hand. Fruits were placed in plastic boxes and immediately returned to the laboratory of the Guizhou fruit processing engineering technology research center. Next, fruits of uniform size that were free from mechanical damage, plant diseases, and insect pests were selected for the experiment. First, the blueberries were treated with 0, 0.1, 0.3, and 0.5 mmol L^−1^ MT and stored at 0 ± 0.5°C for 80 days. The preliminary test results showed that 0.3 mmol L^−1^ MT treatment significantly inhibited blueberry rot. Therefore, 0.3 mmol L^−1^ MT was used for further study. The fruits were randomly divided into two groups, 150 boxes of fruits in each group, immersed in MT solution (0.3 mmol L^−1^) or distilled water (control) for 3min. Subsequently, the fruits were removed and allowed to dry naturally at room temperature. Finally, all the blueberries were packed in plastic boxes (120 fruits per box), in polyethylene (PE20) bags, and precooled at 0 ± 0.5°C for 48 h and, then, the bag was tied. Every 20 days, some of the fruits were removed from storage at 0 ± 0.5°C, cut into small pieces, frozen in liquid nitrogen, and immediately stored at -80°C. The enzyme activity, gene expression, and phenylpropane metabolites in the fruits were subsequently analyzed.

### Preparation of Spore Suspensions and Determination of Mycelial Growth and Spore Germination

*A. alternata*, *B. cinerea*, and *C. gloeosporioides* were isolated from the diseased blueberries. The purified pathogenic fungi were inoculated into potato dextrose agar (PDA) medium and incubated at 25°C for 7 days. Then, 10 ml (containing 0.01% Tween 80) of sterile water was added to the Petri dishes, and the spore solution was diluted with sterile water by light scraping with a sterilized applicator, followed by the filtration of the solution through four layers of sterile gauze. The spore suspension was adjusted to 1 × 10^6^ spores/mL by using a hemocytometer.

The inhibitory effect of MT on the mycelia of *A. alternata*, *B. cinerea*, and *C. gloeosporioides* was determined using the method reported by Pan et al. (2022). MT was dissolved in a small volume of ethanol and mixed with sterile water. The colonies of *A. alternata*, *B. cinerea*, and *C. gloeosporioides* were punched with a sterile punch (5-mm diameter) from the edge of the colonies cultured for 5 days and placed in the center of the medium containing 0 (blank control) or 0.3 mmol L^−1^MT in PDA (containing 0.05% ethanol by volume). The colonies were incubated at 25°C for 6 days, and the colony diameters were measured every 3 days. Each treatment was repeated three times.

The spore germination assay was slightly modified from the method described by Olmedo et al. (2017). Sterilized slides were placed in PDA medium containing 0 or 0.3 mmol L^−1^ MT, and after the medium had solidified, 5 μL of *A. alternata*, *B. cinerea*, and *C. gloeosporioides* spore solutions were placed in the center of the slides. The slides were placed in sterile Petri dishes and incubated at 25°C with 80–85% relative humidity (RH) for 8 h. Spore germination was observed at different times (after 4 and 8 h) using a CX21 light microscope (Olympus Co., Ltd., Tokyo, Japan), and each treatment was repeated three times. The spores were considered to have germinated when the length of the spore germination tube was greater than the diameter of the spore itself, and 100 spores were observed for each treatment to calculate the germination rate. The germination rate was given by the number of germinated spores divided by the total number of spores.

Determination of PAL, C4H, 4CL, CAD, PPO, and POD activities.

The PAL activity in the blueberries was determined using the method described by Zhang W. et al. (2021) with appropriate modifications. Briefly, 0.5 ml enzyme solution, 0.5 ml of 20 mmol/L l-phenylalanine solution, and 3.0 ml of 50 mmol L^−1^ borate buffer (pH 8.8, containing 5 mmol L^−1^ *β*-mercaptoethanol, 2 mmol L^−1^ of ethylenediaminetetraacetic acid (EDTA), and 40 g L^−1^ polyvinylpyrrolidone (PVP)) were added to the reaction tubes. After placing the reaction tube in a water bath heated to 37°C for 60 min, 0.1 ml of 6 mol L^−1^ HCl was added to terminate the reaction, and the absorbance value of the reaction solution was measured at 290 nm. A change in the optical density (OD, 0.01 h^−1^) was taken as a unit of the enzyme activity, which is expressed in U g^−1^, where U = 0.01ΔOD_290_ h^−1^.

The C4H and 4CL activities in the blueberries were determined using the method described by Li et al. (2019) with appropriate modifications. For C4H, 50 μL of enzyme solution, 100 μL of 0.5 mmol L^−1^ d-glucose-6-phosphate disodium salt, 1 ml of 2 mmol L^−1^ *trans*-cinnamic acid, 100 μL of 0.5 mmol L^−1^ oxidized disodium coenzyme II, and 2 ml of 50 mmol L^−1^ extraction buffer (pH 7.5, containing 2 mmol L^−1^ of *β*-mercaptoethanol) were added to the reaction tube. After heating the reaction tube in a water bath at 37°C for 1 h, the reaction was terminated by adding 200 μL of 6 mol L^−1^ HCL, and the absorbance of the reaction solution was measured at 340 nm. The change in the OD value (0.01 min^−1^) was used as a unit of enzyme activity, which was expressed in U g^−1^, where U = 0.01ΔOD_340_ min^−1^.

For 4CL, the reaction system was 0.1 ml of enzyme solution, 0.3 ml of 50 μmol L^−1^ adenosine triphosphate (ATP), 0.3 ml of 5 μmol L^−1^ coenzyme A (CoA), and 0.15 ml of 0.3 μmol L^−1^ *p*-coumaric acid. The reaction solution was heated in a water bath at 40°C for 10 min, and the absorbance of the reaction solution was measured at 333 nm. The change in the OD value (0.01 min^−1^) was used as a unit of the enzyme activity, which is expressed in U g^−1^, where U = 0.01ΔOD_333_ min^−1^.

The CAD activity in the blueberries was determined by using the method of Li et al. (2017) with appropriate modifications. Briefly, 0.5 ml of enzyme solution, 1.4 ml of 1 mol L^−1^ *trans*-cinnamic acid, and 1 ml of 2 mmol L^−1^ nicotinamide adenine dinucleotide phosphate (NADPH) were added to the reaction tube. The reaction tube was kept in a water bath at 37°C for 1 h, and then, the reaction was terminated by adding 0.2 ml of 1 mol L^−1^ HCl. The absorbance of the reaction solution was measured at a wavelength of 340 nm. The change in the OD value (0.01 min^−1^) was used as a unit of the enzyme activity, which is expressed in U g^−1^.

The method of Lin et al. (2013) with slight modifications was used to determine the PPO activity in the blueberries. First, 3 ml of the enzyme extract was added to 3.9 ml of the 0.05 mol L^−1^ sodium phosphate buffer solution and 1 ml of 0.1 mol L^−1^ catechol solution. The reaction solution was kept in a water bath at 37°C for 10 min, and 2 ml of 20% trichloroacetic acid was then added to terminate the reaction. The absorbance of the reaction solution was recorded at a wavelength of 420 nm. One unit of the PPO activity was defined as the amount of enzyme required to increase the OD value by 0.01 min^−1^, and the results are expressed in U g^−1^.

The POD activity in the blueberries was determined using the method of Wang et al. (2021). Briefly, 0.5 ml of the enzyme extract was added to 3.0 ml of a mixture of 25 mmol L^−1^ guaiacol and 200 μL of 0.5 mol L^−1^ H_2_O_2_, and the change in absorbance at 470 nm was immediately recorded. The change in the OD value (1 min^−1^) was used as a unit of the enzyme activity, which is expressed in U g^−1^.

### Determination of the Contents of Phenylpropanol Metabolites

The total phenolic content of the blueberries was determined using the forintanol method (Liu et al., 2018). Briefly, a sample (2 g) was homogenized with 70% ethanol, centrifuged at 4°C and 10,000 rpm for 15 min, and the supernatant was collected. The supernatant (1 ml) and 3 ml of 0.25 mol L^−1^ of Folin phenol were added to a 25-ml volumetric flask and shaken. After allowing it to stand for 30 s, 6 ml of 12% sodium carbonate solution was added, and distilled water was added to make the solution up to the scale line. It was then left for 1 h. Then, the absorbance was measured at 765 nm. The total phenol content is expressed as milligrams per gram.

The flavonoid content of the blueberries was determined using the method of Wang et al. (2020b). Fruit pulp (1 g) was homogenized with the precooled 1% HCI–methanol solution and transferred to a 20-ml graduated tube to ensure a constant volume. The filtrate was stored at 4°C for 20 min, filtered, and collected. The absorbance was measured at 325 nm, and the flavonoid content is expressed as milligrams per gram.

The anthocyanin content in the blueberries was determined according to the method reported by Chen et al. (2017) with slight modifications. Each sample (2 g) was homogenized by grinding with a small amount of 60% ethanol and made up to 50 ml with 60% ethanol. After incubation in a water bath at 50°C for 60 min, the solution was filtered, and the filtrate was collected. Two test tubes were taken, one of which is added with 1 ml of filtrate and 9 ml of pH KCl buffer solution, and the other is added with 1 ml of the filtrate and 9 ml of pH 4.5 sodium acetate (NaAc) buffer solution. The absorbance values were measured at 510 and 700 nm after standing for 40 min. The anthocyanin content is expressed as milligrams/100 g of the fruit.

The method described by Zhang et al. (2020) with slight modifications was used to determine the lignin content of the blueberries. A sample of 1 g was homogenized by grinding with 5 ml of 95% ethanol, and the precipitate was collected by centrifugation at 4°C and 1,000*g* for 15 min. The precipitate was washed three times with ethanol and hexane (1:2, v/v) and collected. Subsequently, the precipitate was dried in an oven at 60°C. Then, 1 ml of a 25% acetyl bromide–glacial acetic acid solution was added to the dried precipitate, and this mixture was held in a water bath at 70°C for 30 min. Afterward, 1 ml of 2 mol L^−1^ NAOH was added to terminate the reaction. Then, 2 ml of glacial acetic acid and 0.1 ml of 7.5 mol L^−1^ hydroxylamine hydrochloride were added to the reaction solution, which was centrifuged again to collect the supernatant. The absorbance was measured at 280 nm. The lignin content is expressed as OD_280_ g^−1^ fresh weight (FW).

### Quantitative Real-Time Polymerase Chain Reaction (qRT-PCR) Analysis

Blueberry memory RNA (mRNA) was extracted using a Total Plant RNA Extraction Kit (TIANGEN Biotech Co., Ltd., Beijing, China). The mRNA concentration and purity were determined using a NanoDrop2000 ultra-micro spectrophotometer (Thermo Scientific, United States), and its integrity was examined by 1% agarose gel electrophoresis. Copy DNA (cDNA) was generated by the reverse transcription of RNA using the Servicebio® RT First Strand cDNA Synthesis Kit (Servicebio, United States) and subjected to fluorescent qRT-PCR. The primer sequences used are listed in Table 1, and *VaMET* was used as the internal reference gene (Wang et al., 2021). The relative gene expression was calculated using the 2^−Δ^ ^Δ Ct^ method (Naik et al., 2007).

**TABLE 1:** Primer sequences for fluorescence qRT-PCR.

Gene	Forward primer (5′→3′)	Reverse primer (5′→3′)
*VaMET*	ACC​CTG​ACA​TGA​GCT​TCT​CG	ACC​CAA​ATC​TCT​GCT​TGC​TG
*VaPAL*	AGT​CAT​CCG​ATC​ATC​GAC​AAA​G	TTG​TCC​ATC​GAG​ACT​CCA​ATG
*VaC4H*	GCC​GTT​TCT​CAG​AGG​GTA​TTT​G	CAT​TTC​AGA​CTG​TTG​TTG​TCC​ATC
*Va4CL*	TCT​TAC​TCC​GAC​AAA​CCC​GC	TGA​TAC​CCA​GTT​GGT​TGA​TGA​AG
*VaCAD*	AGG​GAT​AAA​CTT​GGA​GGG​TTT​G	TTG​AAG​CCT​TGA​GCA​TTG​GAA​C
*VaPPO*	GCC​ATT​CTG​GAA​CTG​GGA​CTC	GGT​TGC​TGT​TTA​TTC​GTG​CCT​T
*VaPOD*	GAC​CTG​AAG​TCC​CAT​TCC​ATC	ATC​CAG​AAC​GCT​CCT​TGT​GG
*VaGLU*	GCC​GTT​GGG​AAT​GAA​GTG​AAT	GGA​ATG​GGT​TGC​TTA​CGA​GTG
*VaCHT*	GGT​AGC​AAC​TGA​CCC​AAC​CAT​T	GAT​CCC​ACC​GTT​GAT​TAT​GTT​AGT
*VaLOX*	GGA​AAG​CCA​CAG​TGG​AAG​CA	AGA​GTC​GTG​AGC​GAG​GAC​ATG
*VaAOS*	GAT​CAA​GCC​GCG​TTC​AAT​T	CGG​AGG​GAT​ACG​GAA​GGT​GT
*VaAOC*	ACT​CAG​AGC​CAT​CTC​CTC​CG	TTC​TGT​TGG​GGT​GCT​CTG​G

### Statistical Analysis

All the experiments were repeated three times, and the data are expressed as the mean ± standard deviation (SD). Duncan’s multiple range test was used for the analysis, and significance was set at *p* < 0.05. Origin 2018 was used to plot the data.

## Results

Effects of melatonin treatment on growth inhibition and spore germination of *A. alternata*, *B. cinerea*, and *C. gloeosporioides in vitro*.

**FIGURE 1:**
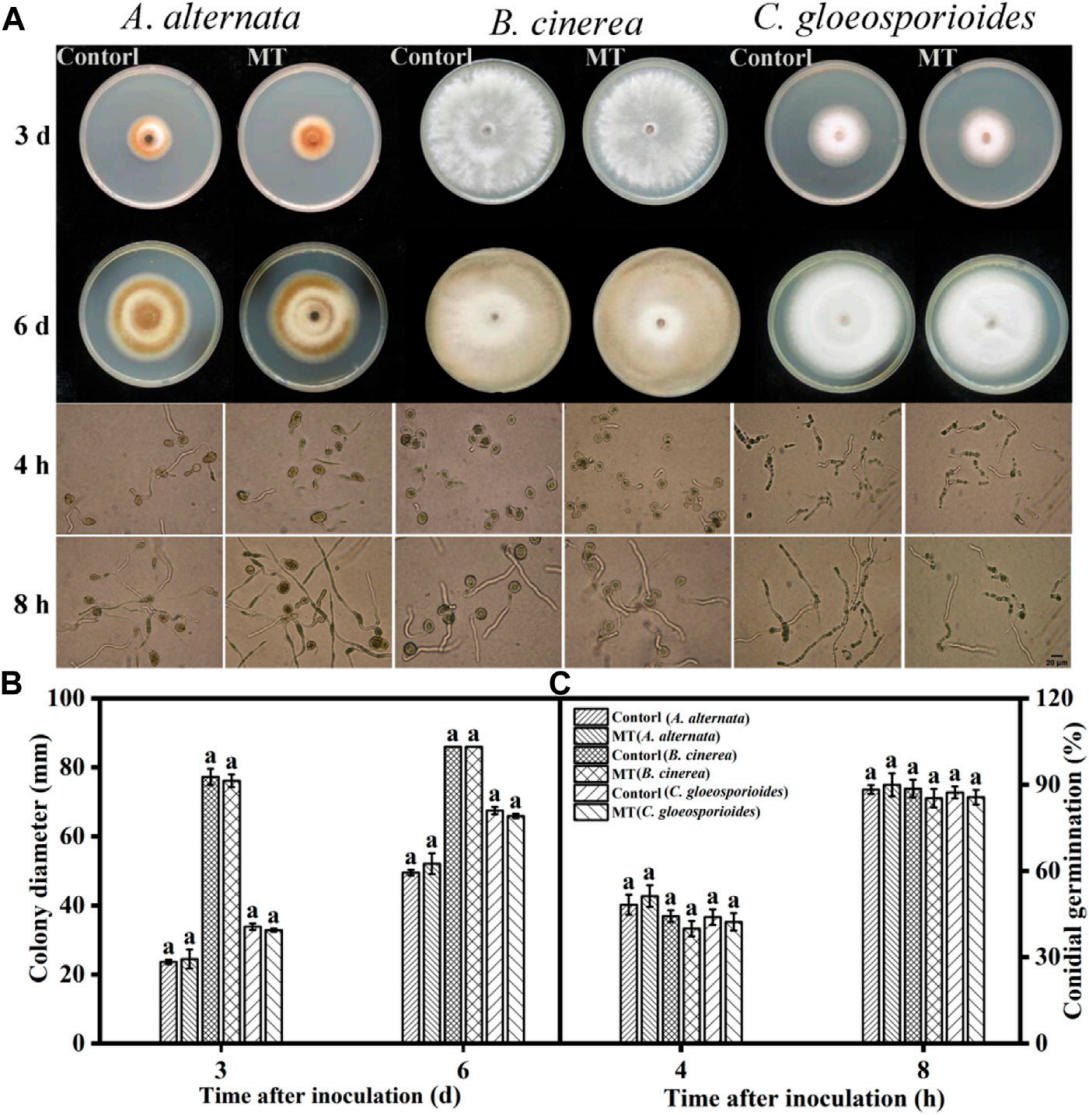
Effects of MT on *in vitro* growth inhibition and spore germination of *A. alternata*, *B. cinerea*, and *C. gloeosporoides*: **(A)** Photographs of cultures, **(B)** colony diameters, and **(C)** spore germination rates. Significant differences between the MT-treated and control groups were compared for the same strain. Different letters represent significant differences (*p* < 0.05). Data are presented as the mean ± SD.

As shown in Figures 1A,B, there was no significant difference (*p* > 0.05) in the diameters of the colonies of *A. alternata*, *B. cinerea*, and *C. gloeosporioides* after MT treatment compared to the control group after 3 and 6 days. Furthermore, the spore germination rates of the three fungi after MT treatment were not significantly different (*p* > 0.05) from those of the control group. In addition, there was no significant difference in the spore germination rates of the three fungi after MT treatment compared to the control group after 4 and 8 h (*p* > 0.05) (Figure 1C). As shown previously, MT showed no inhibitory activity against *A. alternata*, *B. cinerea*, and *C. gloeosporioides*.

Effect of melatonin treatment on gene expression related to the JA signaling pathway.

**FIGURE 2:**
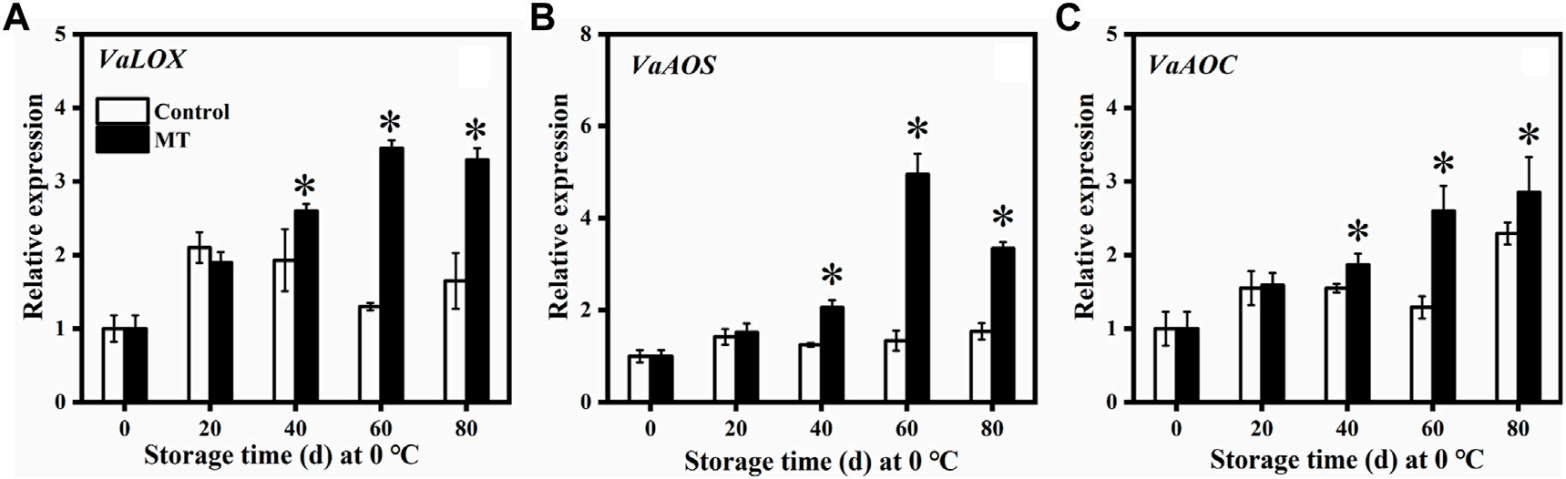
Effects of MT treatment on the gene expressions of *VaLOX* **(A)**, *VaAOS* **(B)**, and *VaAOC* **(C)** of blueberry fruits during the postharvest storage at 0°C. Here, symbol * indicates significant differences (*p* < 0.05). Data are presented as the mean ± SD.

As shown in Figures 2A,B, the relative expression of key genes related to JA synthesis, *VaLOX*, and *VaAOS* (which are related to lipoxygenase (LOX) and allene oxide synthase (AOS)) in the MT-treated fruits first increased and then decreased, reaching the highest relative expression after 60 days (*p* < 0.05), and these were 2.65 and 3.69 times higher than those of the control group, respectively. The relative expression of *VaAOC* in the control fruit was relatively stable in the first 60 days but increased rapidly after 60 days. The relative expression of *VaAOC* (related to allene oxide cyclase (AOC)) in the MT-treated fruit increased gradually from 0 to 80 days and was significantly higher than that in the control group at 40, 60, and 80 days (*p* < 0.05).

### Effect of Melatonin Treatment on the Activities of Defense Enzymes

**FIGURE 3:**
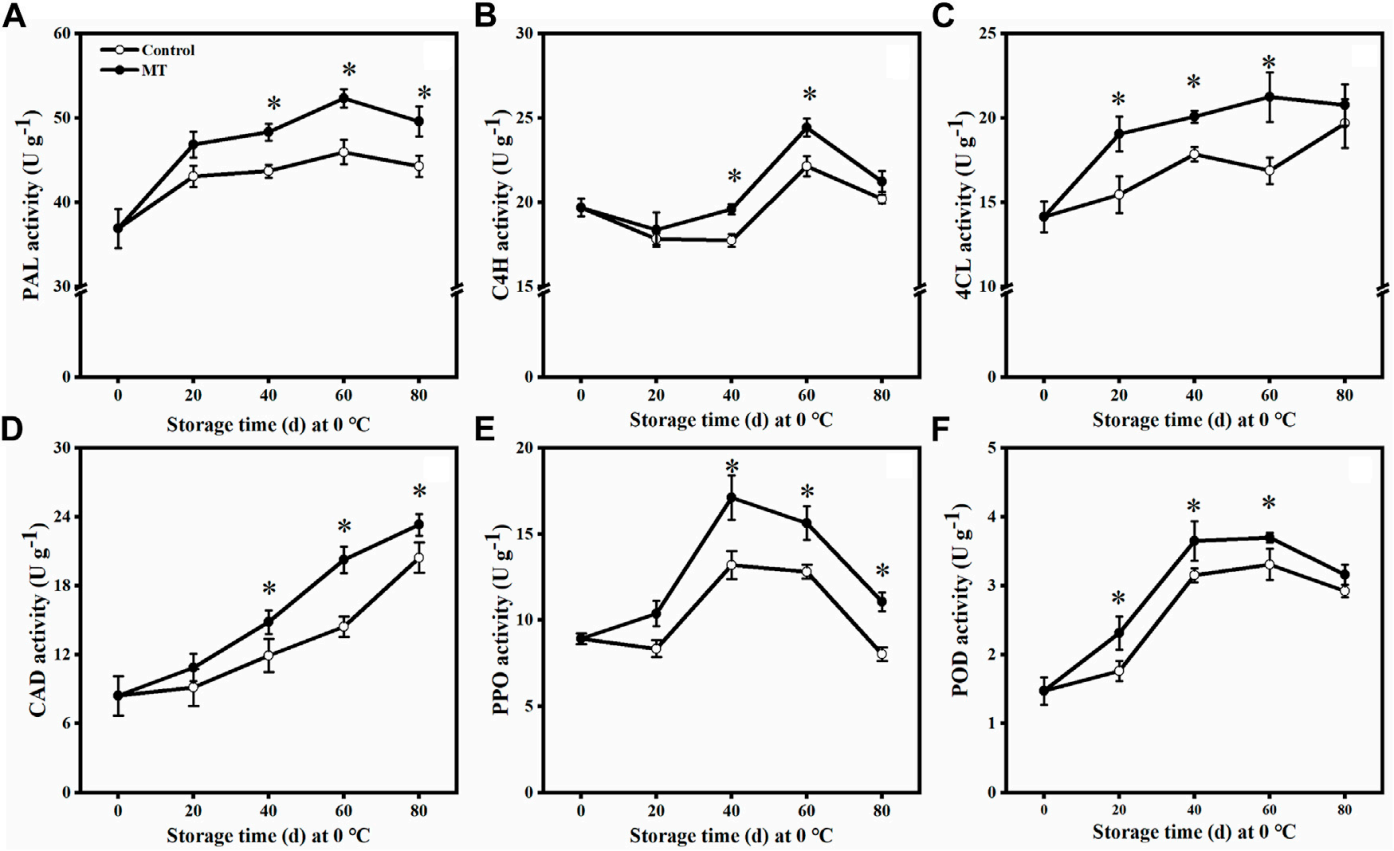
Effects of MT treatment on PAL **(A)**, C4H **(B)**, 4CL **(C)**, CAD **(D)**, PPO **(E)**, and POD**(F)** activities of blueberry fruits during the postharvest storage at 0°C. Here, symbol * indicates significant differences (*p* < 0.05). Data are presented as the mean ± SD.

As shown in Figures 3A,C,E,F, the activities of PAL, 4CL, PPO, and POD in the MT-treated fruits increased and then decreased from 0 to 80 days. The PAL and 4CL activities were the highest after 60 days (*p* < 0.05), being 1.14 and 1.26 times higher than those of the control group, respectively. The activities of PPO and POD were the highest after 40 days (*p* < 0.05), being 1.30 and 1.16 times higher than those of the control group, respectively.

As shown in Figure 3B, the C4H activity decreased slightly during the first 20 days. After 40 and 60 days, the C4H activity in the MT-treated group was significantly higher than that in the control group (*p* < 0.05), being 1.10 and 1.11 times higher than that in the control group, respectively. The CAD activity of both the MT-treated and control fruits gradually increased from 0 to 80 days (Figure 3D). In particular, MT treatment significantly increased the CAD activity after 40, 60, and 80 days (*p* < 0.05), and these values are 1.25, 1.40, and 1.14 times higher than those of the control group, respectively.

### Effect of Melatonin Treatment on the Expression of Genes Related to Defense Enzymes

**FIGURE 4:**
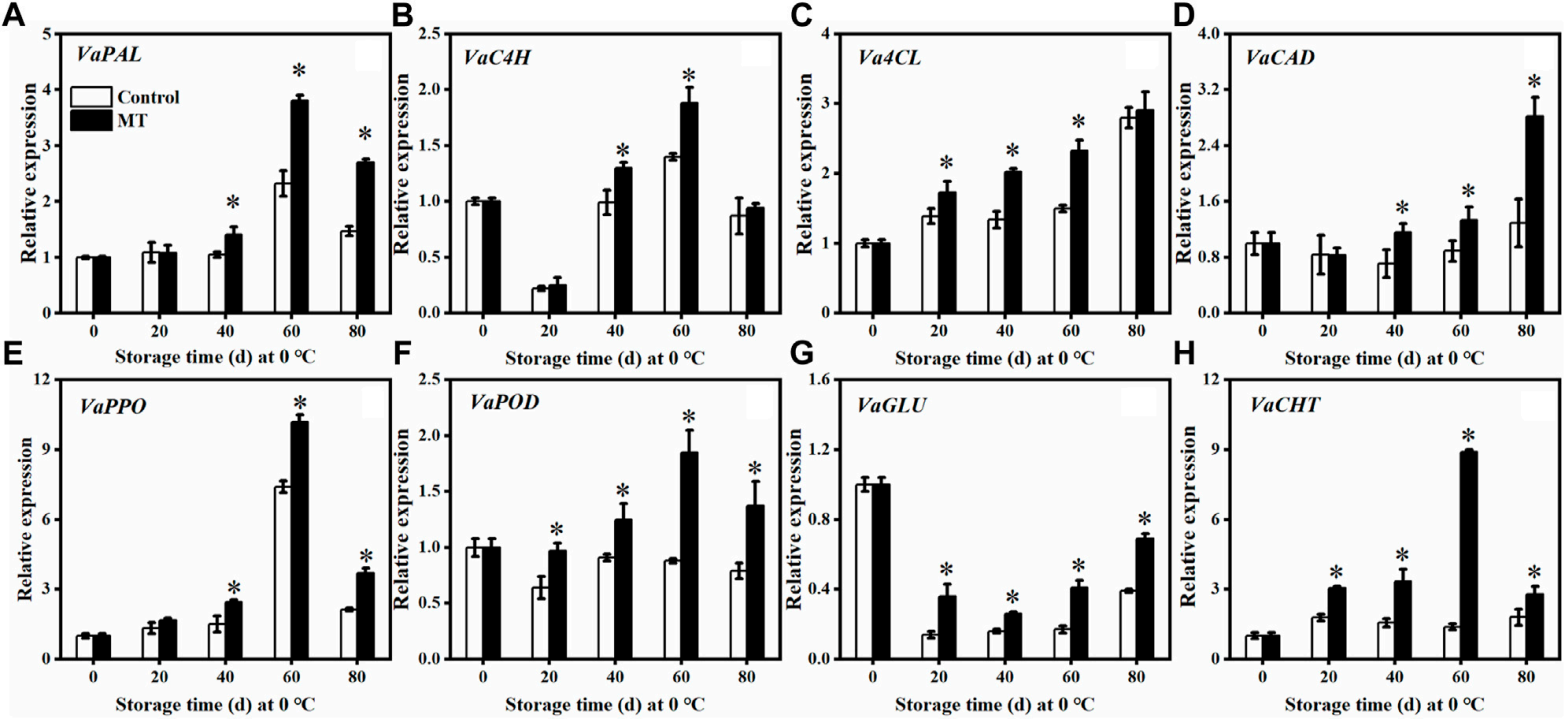
Effects of MT treatment on the gene expressions of *VaPAL* **(A)**, *VaC4H* **(B)**, *Va4CL* **(C)**, *VaCAD* **(D)**, *VaPPO* **(E)**, *VaPOD* **(F)**, *VaGLU* **(G)**, and *VaCHT* **(H)** during the postharvest storage at 0°C. Here, symbol * indicates significant differences (*p* < 0.05). Data are presented as the mean ± SD.

As shown in Figures 4A–D, the relative expressions of *VaPAL* and *VaC4H* in the MT-treated fruits were not significantly different from those of the control group during the first 20 days (*p* > 0.05). After 60 days of storage, the relative expressions of *VaPAL* and *VaC4H* reached the highest levels (*p* < 0.05), which are 1.70 and 1.34 times higher than those of the control group, respectively. In contrast, the relative expressions of *Va4CL* and *VaCAD* in the MT-treated fruits reached the highest level after 80 days: 1.04 and 2.19 times higher than that of the control group, respectively. Compared with the control group, the relative expression of *Va4CL* in the MT-treated fruits was significantly higher (*p* < 0.05) after 20, 40, and 60 days, whereas the relative expression of *VaCAD* was significantly higher (*p* < 0.05) after 40 days. In contrast, the relative expressions of *VaPPO* (Figure 4E) and *VaPOD* (Figure 4F) in the MT-treated fruits increased and then decreased. After 40 days of storage, both *VaPPO* and *VaPOD* expressions were significantly upregulated after MT treatment (*p* < 0.05).

Concerning the pathogenesis-related proteins, the relative expression of *VaGLU* in the MT-treated group and the control group decreased and then increased during storage (Figure 4G). Overall, the relative expression of *VaGLU* was low but was significantly increased after MT treatment (*p* < 0.05). Throughout the storage period, the relative expression of *VaCHT* in the MT-treated fruits was significantly (*p* < 0.05) higher than that in the control (Figure 4H), specifically, 1.71, 2.13, 6.40, and 1.56 times higher than that in the control group after 0, 20, 40, 60, and 80 days, respectively.

### Effects of Melatonin Treatment on the Total Phenol, Flavonoid, Anthocyanin, and Lignin Contents in the Blueberry Fruit

**FIGURE 5:**
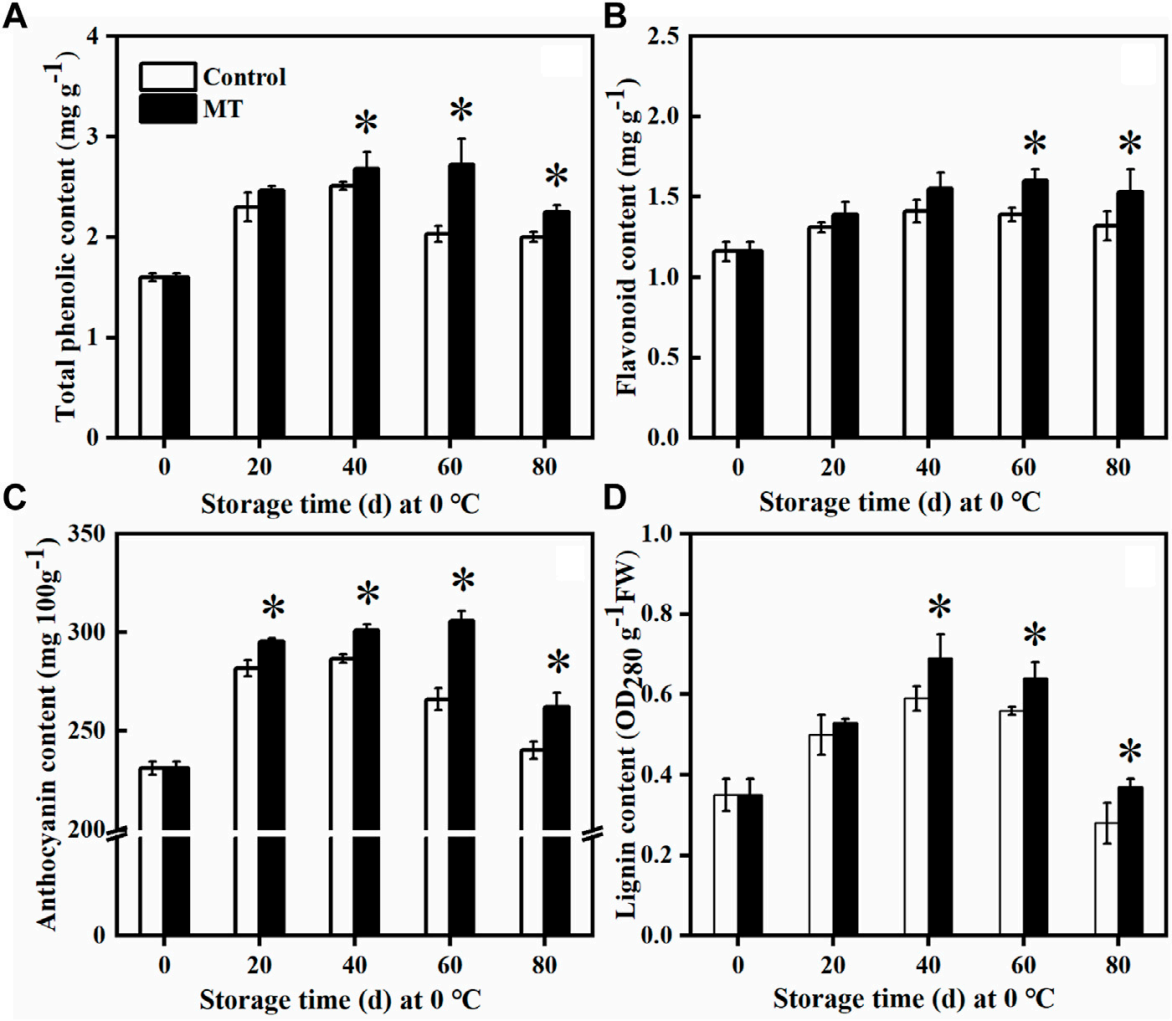
Effects of MT treatment on the total phenol **(A)**, flavonoid **(B)**, anthocyanin **(C)**, and lignin **(D)** contents of blueberry fruits during postharvest storage at 0°C. Here, symbol * indicates significant differences (*p* < 0.05). Data are presented as the mean ± SD.

As shown in Figure 5, MT treatment promoted the accumulation of total phenols, flavonoids, anthocyanins, and lignin in the blueberries. After 60 days of storage, the total phenolic content of the MT-treated fruits reached the highest level (*p* < 0.05), which is 1.34 times higher than that of the control group (Figure 5A). The initial flavonoid content of the fruit was 1.16 mg g^−1^, which gradually increased from 0 to 60 days and slightly decreased after 80 days of storage. The flavonoid content of the MT-treated fruit was significantly higher than that of the control group after 60 and 80 days of storage (*p* < 0.05) (Figure 5B). Throughout the storage period, the anthocyanin and lignin contents increased and then decreased, and after 20 days of storage, the anthocyanin content of the MT-treated fruits was significantly higher than that of the control group (*p* < 0.05): 1.04, 1.05, 1.02, and 1.09 times higher than that of the control group (Figure 5C), respectively. The lignin content of the MT-treated fruits reached the highest level after 40 days (*p* < 0.05), and this was 10.00% higher than that of the control group, decreasing to a lower level after 80 days. Therefore, MT treatment promoted the accumulation of polyphenols, flavonoids, anthocyanins, and lignin in the fruit and delayed their decay in the late storage period, suggesting improvement in the disease resistance of the blueberries.

## Discussion

JA is a signaling molecule that activates plant defenses to counter pathogen infestation and is synthesized in plants mainly through the octadecenoic acid pathway (Ruan et al., 2019). In this pathway, LOX are the key enzymes that convert linolenic acid to 13(*S*)-hydroxylinolenic acid, which is further converted by AOS and AOC to 12-oxo-phytodienoic acid. This product is then reduced and *β*-oxidized to produce JA (Ruan et al., 2019). It has been shown that JA enhances the defenses of peaches against *Penicillium expansum* by inducing NO production and, thus increasing the PAL, GLU, and CHT enzyme activities (Yu et al., 2012). In addition, JA induces the upregulation of *PAL* and *CHT* gene expressions and promotes phenolic product accumulation in tobacco plants (Keinnen et al., 2001). The results of this study showed that MT treatment upregulated *VaLOX*, *VaAOS*, and *VaAOC* gene expressions (Figure 2) and promoted JA synthesis, which in turn induced the accumulation of disease resistance–inducing substances in the blueberry fruits (Figure 5). This result is consistent with the study of Liu et al. (2019), who applied exogenous MT to induce improved resistance to *B. cinerea* in tomatoes.

When plants are exposed to biotic or abiotic stresses, a series of defense reactions are initiated. Phenylpropane metabolism is an important defense response pathway in the plant body (Wei et al., 2017). PAL is a key rate-limiting enzyme in the metabolism of phenylpropane and catalyzes the formation of cinnamic acid from phenylpropane, which is further converted to *p*-coumaric acid by C4H (Hu et al., 2014). POD and CAD are involved in lignin synthesis, whereas PPO is closely associated with plant resistance to pathogenic microbial invasion, specifically by catalyzing the oxidation of phenolic substances to produce antimicrobial substances (Hu et al., 2019). Li et al. (2019) found that MT treatment increased the activities of enzymes related to phenylpropane metabolism and enhanced the resistance of tomato fruits to *B. cinerea*. Zhang H. et al. (2021) demonstrated that salicylic acid induces the elevated expression of genes for enzymes related to phenylpropane metabolism, promotes the accumulation of phenylpropane secondary metabolites, and reduces the postharvest rot in *Lycium barbarum* fruit. Liu et al. (2022) showed that methionine treatment promotes lignin accumulation and enhances the resistance of jujube to *A. alternata.* In this study, MT treatment increased the activities of enzymes related to phenylpropane metabolism (PAL, C4H, 4CL, CAD, PPO, and POD) (Figure 3), the expressions of related genes (*VaPAL*, *VaC4H*, *Va4CL*, *VaCAD*, *VaPPO*, and *VaPOD*) (Figure 4), and the accumulation of polyphenols, flavonoids, anthocyanins, and lignin (Figure 5), thereby improving the postharvest disease resistance in blueberries.

GLU and CHT are pathogenesis-related proteins in plant tissues that can induce resistance to further invasion by pathogenic bacteria, disrupt the cell walls of fungi, and reduce fungal viability, thereby enhancing disease resistance (Hu et al., 2019). Huang et al. (2021) found that MT treatment upregulated *GLU* and *CHT* gene expressions and enhanced the resistance of ginger rootstocks to *Fusarium oxysporum* and *Penicillium compactum*. In our study, we found that MT treatment upregulated the expression of the pathogenesis-related proteins, *VaGLU* and *VaCHT* (Figures 4G,H), which is consistent with the abovementioned results. In addition, we found that MT did not significantly inhibit the colony diameter or spore germination of *A. alternata*, *B. cinerea*, and *C. gloeosporioides* (Figure 1). This result is consistent with the results of Li et al. (2019), who used MT treatment to induce resistance to *B. cinerea* in tomatoes, and Zhang et al. (2022), who applied MT treatment to induce resistance to *A. alternata* in jujube fruit. In summary, MT had no inhibitory activity against *A. alternata*, *B. cinerea*, and *C. gloeosporioides*, suggesting that it reduced the postharvest rot of blueberries by inducing the activation of the JA signaling pathway and regulating the accumulation of phenylpropane metabolites.

## Conclusion

In summary, the results of this study showed that MT treatment promoted the accumulation of polyphenols, flavonoids, anthocyanins, and lignans. In addition, MT treatment increased the enzymatic activities of PAL, C4H, 4CL, CAD, PPO, and POD and upregulated the expression of genes related to JA synthesis (*VaLOX*, *VaAOS,* and *VaAOC*), pathogenesis-related proteins (*VaGLU* and *VaCHT*), and genes related to phenylpropane metabolism (*VaPAL*, *VaC4H*, *Va4CL*, *VaCAD*, *VaPPO*, and *VaPOD*). These results suggest that MT treatment of blueberries may induce the activation of JA signaling and phenylpropane pathways as defense responses, thus effectively controlling blueberry rot during storage. Therefore, the application of MT could be an effective strategy for preserving blueberries after harvest.

## Data Availability

The authors acknowledge that the data presented in this study must be deposited and made publicly available in an acceptable repository, prior to publication. Frontiers cannot accept an article that does not adhere to our open data policies.

## References

[B2] Bennett R. N. Wallsgrove R. M. (1994). Secondary Metabolites in Plant Defence Mechanisms. New Phytol. 127, 617–633. 10.1111/j.1469-8137.1994.tb02968.x 33874382

[B3] Bhardwaj R. Pareek S. Saravanan C. Yahia E. M. (2022). Contribution of Pre-storage Melatonin Application to Chilling Tolerance of Some Mango Fruit Cultivars and Relationship with Polyamines Metabolism and γ-aminobutyric Acid Shunt Pathway. Environ. Exp. Bot. 194, 104691. 10.1016/j.envexpbot.2021.104691

[B4] Chen Y. Hung Y.-C. Chen M. Lin H. (2017). Effects of Acidic Electrolyzed Oxidizing Water on Retarding Cell Wall Degradation and Delaying Softening of Blueberries during Postharvest Storage. Lwt 84, 650–657. 10.1016/j.lwt.2017.06.011

[B7] Gao S. Ma W. Lyu X. Cao X. Yao Y. (2020). Melatonin May Increase Disease Resistance and Flavonoid Biosynthesis through Effects on DNA Methylation and Gene Expression in Grape Berries. BMC Plant Biol. 20 (1), 231. 10.1186/s12870-020-02445-w 32448301 PMC7247213

[B8] Ge Y. Li X. Li C. Tang Q. Duan B. Cheng Y. (2019). Effect of Sodium Nitroprusside on Antioxidative Enzymes and the Phenylpropanoid Pathway in Blueberry Fruit. Food Chem. 295, 607–612. 10.1016/j.foodchem.2019.05.160 31174802

[B9] Gong P. Chen F.-x. Wang L. Wang J. Jin S. Ma Y.-m. (2014). Protective Effects of Blueberries (Vaccinium Corymbosum L.) Extract against Cadmium-Induced Hepatotoxicity in Mice. Environ. Toxicol. Pharmacol. 37, 1015–1027. 10.1016/j.etap.2014.03.017 24751684

[B11] Hu M. Yang D. Huber D. J. Jiang Y. Li M. Gao Z. (2014). Reduction of Postharvest Anthracnose and Enhancement of Disease Resistance in Ripening Mango Fruit by Nitric Oxide Treatment. Postharvest Biol. Technol. 97, 115–122. 10.1016/j.postharvbio.2014.06.013

[B12] Hu M. Zhu Y. Liu G. Gao Z. Li M. Su Z. (2019). Inhibition on Anthracnose and Induction of Defense Response by Nitric Oxide in Pitaya Fruit. Sci. Hortic. 245, 224–230. 10.1016/j.scienta.2018.10.030

[B13] Huang K. Sui Y. Miao C. Chang C. Wang L. Cao S. (2021). Melatonin Enhances the Resistance of Ginger Rhizomes to Postharvest Fungal Decay. Postharvest Biol. Technol. 182, 111706. 10.1016/j.postharvbio.2021.111706

[B14] Ji N. Wang J. Li Y. Li M. Jin P. Zheng Y. (2021a). Involvement of PpWRKY70 in the Methyl Jasmonate Primed Disease Resistance against Rhizopus Stolonifer of Peaches via Activating Phenylpropanoid Pathway. Postharvest Biol. Technol. 174, 111466. 10.1016/j.postharvbio.2021.111466

[B15] Ji Y. Hu W. Liao Z. Jiang A. Yang X. Guan Y. (2021b). Effect of Ethanol Vapor Treatment on the Growth of *Alternaria alternata* and *Botrytis Cinerea* and Defense-Related Enzymes of Fungi-Inoculated Blueberry during Storage. Front. Microbiol. 12, 618252. 10.3389/fmicb.2021.618252 33574808 PMC7870470

[B16] Keinnen M. Oldham N. J. Baldwin I. T. (2001). Rapid HPLC Screening of Jasmonate-Induced Increases in Tobacco Alkaloids, Phenolics, and Diterpene Glycosides in Nicotiana Attenuata. J. Agric. Food Chem. 49 (8), 3553–3558. 10.1021/jf01020010.1021/jf010200+ 11513627

[B17] Li H. Suo J. Han Y. Liang C. Jin M. Zhang Z. (2017). The Effect of 1-methylcyclopropene, Methyl Jasmonate and Methyl Salicylate on Lignin Accumulation and Gene Expression in Postharvest 'Xuxiang' Kiwifruit during Cold Storage. Postharvest Biol. Technol. 124, 107–118. 10.1016/j.postharvbio.2016.10.003

[B18] Li S. Xu Y. Bi Y. Zhang B. Shen S. Jiang T. (2019). Melatonin Treatment Inhibits Gray Mold and Induces Disease Resistance in Cherry Tomato Fruit during Postharvest. Postharvest Biol. Technol. 157 (C), 110962. 10.1016/j.postharvbio.2019.110962

[B19] Li Z. Wang N. Wei Y. Zou X. Jiang S. Xu F. (2020). Terpinen-4-ol Enhances Disease Resistance of Postharvest Strawberry Fruit More Effectively Than Tea Tree Oil by Activating the Phenylpropanoid Metabolism Pathway. J. Agric. Food Chem. 68, 6739–6747. 10.1021/acs.jafc.0c01840 32379969

[B20] Lin Y.-F. Hu Y.-H. Lin H.-T. Liu X. Chen Y.-H. Zhang S. (2013). Inhibitory Effects of Propyl Gallate on Tyrosinase and its Application in Controlling Pericarp Browning of Harvested Longan Fruits. J. Agric. Food Chem. 61, 2889–2895. 10.1021/jf305481h 23427826

[B21] Lin Y. Huang G. Zhang Q. Wang Y. Dia V. P. Meng X. (2020). Ripening Affects the Physicochemical Properties, Phytochemicals and Antioxidant Capacities of Two Blueberry Cultivars. Postharvest Biol. Technol. 162, 111097. 10.1016/j.postharvbio.2019.111097

[B22] Lin Y. Wang Y. Li B. Tan H. Li D. Li L. (2018). Comparative Transcriptome Analysis of Genes Involved in Anthocyanin Synthesis in Blueberry. Plant Physiology Biochem. 127, 561–572. 10.1016/j.plaphy.2018.04.034 29727860

[B23] Liu C. Chen L. Zhao R. Li R. Zhang S. Yu W. (2019). Melatonin Induces Disease Resistance to *Botrytis Cinerea* in Tomato Fruit by Activating Jasmonic Acid Signaling Pathway. J. Agric. Food Chem. 67 (22), 6116–6124. 10.1021/acs.jafc.9b00058 31084000

[B24] Liu C. Zheng H. Sheng K. Liu W. Zheng L. (2018). Effects of Postharvest UV-C Irradiation on Phenolic Acids, Flavonoids, and Key Phenylpropanoid Pathway Genes in Tomato Fruit. Sci. Hortic. 241, 107–114. 10.1016/j.scienta.2018.06.075

[B25] Liu Y. Lei X. Deng B. Chen O. Deng L. Zeng K. (2022). Methionine Enhances Disease Resistance of Jujube Fruit against Postharvest Black Spot Rot by Activating Lignin Biosynthesis. Postharvest Biol. Technol. 190, 111935. 10.1016/j.postharvbio.2022.111935

[B26] Moustafa-Farag M. Almoneafy A. Mahmoud A. Elkelish A. Arnao M. Li L. (2020). Melatonin and its Protective Role against Biotic Stress Impacts on Plants. Biomolecules 10, 54. 10.3390/biom10010054 PMC7022677 31905696

[B27] Naik D. Dhanaraj A. L. Arora R. Rowland L. J. (2007). Identification of Genes Associated with Cold Acclimation in Blueberry (Vaccinium Corymbosum L.) Using a Subtractive Hybridization Approach. Plant Sci. 173, 213–222. 10.1016/j.plantsci.2007.05.003

[B28] Olmedo G. M. Cerioni L. González M. M. Cabrerizo F. M. Rapisarda V. A. Volentini S. I. (2017). Antifungal Activity of β-carbolines on Penicillium digitatum and Botrytis Cinerea. Food Microbiol. 62, 9–14. 10.1016/j.fm.2016.09.011 27889171

[B29] Pan L. Chen X. Xu W. Fan S. Wan T. Zhang J. (2022). Methyl Jasmonate Induces Postharvest Disease Resistance to Decay Caused by *Alternaria alternata* in Sweet Cherry Fruit. Sci. Hortic. 292, 110624. 10.1016/j.scienta.2021.110624

[B30] Ren Y. Xue Y. Tian D. Zhang L. Xiao G. He J. (2020). Improvement of Postharvest Anthracnose Resistance in Mango Fruit by Nitric Oxide and the Possible Mechanisms Involved. J. Agric. Food Chem. 68 (52), 15460–15467. 10.1021/acs.jafc.0c04270 33320657

[B31] Ruan J. Zhou Y. Zhou M. Yan J. Khurshid M. Weng W. (2019). Jasmonic Acid Signaling Pathway in Plants. Ijms 20 (10), 2479. 10.3390/ijms20102479 PMC6566436 31137463

[B32] Saito S. Wang F. Xiao C.-L. (2022). Natamycin as a Postharvest Treatment to Control Gray Mold on Stored Blueberry Fruit Caused by Multi-Fungicide Resistant *Botrytis Cinerea* . Postharvest Biol. Technol. 187, 111862. 10.1016/j.postharvbio.2022.111862

[B33] Shang F. Liu R. Wu W. Han Y. Fang X. Chen H. (2021). Effects of Melatonin on the Components, Quality and Antioxidant Activities of Blueberry Fruits. Lwt 147 (5), 111582. 10.1016/j.lwt.2021.111582

[B34] Shi H. Chen Y. Tan D.-X. Reiter R. J. Chan Z. He C. (2015). Melatonin Induces Nitric Oxide and the Potential Mechanisms Relate to Innate Immunity against Bacterial Pathogen Infection in Arabidopsis. J. Pineal Res. 59 (1), 102–108. 10.1111/jpi.12244 25960153

[B35] Truman W. Bennett M. H. Kubigsteltig I. Turnbull C. Grant M. (2007). Arabidopsis Systemic Immunity Uses Conserved Defense Signaling Pathways and Is Mediated by Jasmonates. Proc. Natl. Acad. Sci. U.S.A. 104 (3), 1075–1080. 10.1073/pnas.0605423104 17215350 PMC1783366

[B36] Wang H. Chen Y. Lin H. Lin M. Chen Y. Lin Y. (2020b). 1-Methylcyclopropene Containing-Papers Suppress the Disassembly of Cell Wall Polysaccharides in Anxi Persimmon Fruit during Storage. Int. J. Biol. Macromol. 151, 723–729. 10.1016/j.ijbiomac.2020.02.146 32068065

[B37] Wang H. Cheng X. Wu C. Fan G. Li T. Dong C. (2021). Retardation of Postharvest Softening of Blueberry Fruit by Methyl Jasmonate Is Correlated with Altered Cell Wall Modification and Energy Metabolism. Sci. Hortic. 276, 109752. 10.1016/j.scienta.2020.109752

[B38] Wang H. Kou X. Wu C. Fan G. Li T. (2020a). Methyl Jasmonate Induces the Resistance of Postharvest Blueberry to Gray Mold Caused by Botrytis Cinerea. J. Sci. Food Agric. 100 (11), 4272–4281. 10.1002/jsfa.10469 32378217

[B39] Wang S. Y. Chen C.-T. Yin J.-J. (2010). Effect of Allyl Isothiocyanate on Antioxidants and Fruit Decay of Blueberries. Food Chem. 120, 199–204. 10.1016/j.foodchem.2009.10.007

[B40] Wei Y. Zhou D. Peng J. Pan L. Tu K. (2017). Hot Air Treatment Induces Disease Resistance through Activating the Phenylpropanoid Metabolism in Cherry Tomato Fruit. J. Agric. Food Chem. 65 (36), 8003–8010. 10.1021/acs.jafc.7b02599 28813608

[B41] Yu Q. Chen Q. Chen Z. Xu H. Fu M. Li S. (2012). Activating Defense Responses and Reducing Postharvest Blue Mold Decay Caused by Penicillium expansum in Peach Fruit by Yeast Saccharide. Postharvest Biol. Technol. 74, 100–107. 10.1016/j.postharvbio.2012.07.005

[B42] Zhang H. Liu F. Wang J. Yang Q. Wang P. Zhao H. (2021a). Salicylic Acid Inhibits the Postharvest Decay of Goji Berry (Lycium Barbarum l.) by Modulating the Antioxidant System and Phenylpropanoid Metabolites. Postharvest Biol. Technol. 178, 111558. 10.1016/j.postharvbio.2021.111558

[B43] Zhang L. L. Yu Y. W. Chang L. L. Wang X. J. Zhang S. Y. (2022). Melatonin Enhanced the Disease Resistance by Regulating Reactive Oxygen Species Metabolism in Postharvest Jujube Fruit. J. Food Process. Preserv. 46 (3), e16363. 10.1111/jfpp.16363

[B44] Zhang W. Jiang H. Cao J. Jiang W. (2021b). UV-C Treatment Controls Brown Rot in Postharvest Nectarine by Regulating ROS Metabolism and Anthocyanin Synthesis. Postharvest Biol. Technol. 180, 111613. 10.1016/j.postharvbio.2021.111613

[B45] Zhang X. Zong Y. Li Z. Yang R. Li Z. Bi Y. (2020). Postharvest Pichia Guilliermondii Treatment Promotes Wound Healing of Apple Fruits. Postharvest Biol. Technol. 167, 111228. 10.1016/j.postharvbio.2020.111228

